# An innovative strategy to rapidly inactivate 8.2-log *Enterococcus faecalis* in fresh pineapple juice using cold atmospheric plasma

**DOI:** 10.1038/s41598-021-95452-2

**Published:** 2021-08-06

**Authors:** Farshad Sohbatzadeh, Homayoon Yazdanshenas, Amir-Hossain Soltani, Amir Shabannejad

**Affiliations:** 1 Department of Atomic and Molecular Physics, Faculty of Science, University of Mazandaran, Babolsar, Iran; 2 Plasma Technology Research Core, Faculty of Science, University of Mazandaran, Babolsar, Iran; 3 Department of Cellular and Molecular Biology, Faculty of Science, University of Mazandaran, Babolsar, Iran

**Keywords:** Food microbiology, Design, synthesis and processing

## Abstract

*Enterococcus faecalis* is a life-threatening bacterium that resists high levels of antibiotics or chemical preservatives. In this study, we aimed to investigate the inactivation of *E. faecalis* in fresh pineapple juice (FPJ) with two different cold atmospheric plasmas (CAP) reinforced by H_2_O_2_/H_2_O cold vapor: a plasma jet and a surface dielectric barrier discharge (SDBD). CAP treatments for 300 s with plasma jet and 420 s with SDBD caused an 8.2 log reduction of *E. faecalis*. The concentration of reactive oxygen and nitrogen species induced in FPJ by plasmas was also evaluated. In terms of quality attributes of FPJ, no noticeable color changes (ΔE) were observed. Furthermore, a trifle of loss of organic content such as sugars and organic acids was observed after treatments. These results suggest that our rapid CAP strategy effectively inactivated *E. faecalis* in FPJ with no change of color and negligible effects on other physicochemical properties.

## Introduction

Market demand for safe and high-quality fruit juices is increasing since their pleasant flavors and nutritional benefits are known to consumers. However, the health hazards of contaminated fruit juices are also well documented since the pathogenic microorganisms can grow and duplicate in fruit juices and cause infectious diseases in consumers^1^. The high nutritional richness of fruit juices can also be an attractive environment for microorganisms to proliferate^2^. *Enterococcus faecalis* (ATCC 29212) is a well-known and health-endangering bacteria in agricultural products. These bacteria are non-motile, gram-positive, with the capacity of causing a variety of life-threatening infections among which endocarditis and urinary tract infection are the most common ones^3^. *E. faecalis* strains can grow at a temperature of 60° and resist high levels of many antimicrobial agents^4^. *E. faecalis* strains are known to have irrefutable antibiotic resistance which can contribute to their pathogenicity^5^. Hence, effective methods are required to inactivate this pathogen in fruit juices. Since thermal treatments cannot be used for juices, the Addition of chemical preservative agents is currently a widely used method in food industries^6^. Various preservative agents such as sodium benzoate, calcium propionate, and potassium sorbate are used by commercial producers to prevent microbial spoilage in different food products. Among these chemical preservatives, sodium benzoate is usually added to fruit juices and beverages^7^. High levels of sodium benzoate might prevent microorganisms from spoiling the products but can have negative side effects on human cells as well^7,8^. Sodium benzoate can have cytotoxic effects on human cells and cause damage to different tissues. It has been proved that sodium benzoate can have genotoxic effects on human cells and cause mutation and damage to DNA, and consequently promoting normal cells into cancer cells^9^. On the other hand, *E. faecalis* strains are known to have remarkable tolerance to chemical agents and antibiotics^10^. Disadvantages of traditional preservation methods have created a desire of designing new non-thermal technologies for inactivating microorganisms to upgrade the levels of food safety and quality. These technologies, including intense cold atmospheric-pressure plasma (CAP), rely heavily on combining physical and chemical processes to disable or passivate a variety of microorganisms at low temperatures and short treatment times^2,11–14^. It is a well-known fact that plasma techniques generate reactive oxygen and nitrogen species (RONS) such as electrons, radicals, ions, ozone, atomic nitrogen/oxygen^11,15^. The antimicrobial capacity of cold plasma has been investigated by several scholars before. Most of the reported and suggested mechanisms for the antimicrobial activity of cold plasma are literally based on the generation of reactive oxygen species (ROS), reactive nitrogen species (RNS), and a minor contribution from ultraviolet light (UV)^16^. RNS and ROS seem to be responsible for severe damage to microbial DNA and cell wall^17,18^. There are various methods for cold plasma generation such as surface dielectric barrier discharge (SDBD), plasma jets, coronas, and microwave discharges^2,19^. It has been shown that the RONS generated in plasma are device-dependent. Thus it is rational to conclude that switching devices may not lead to the same outcome. Among the above-mentioned methods, SDBD and plasma jet can present a low-cost and effective antimicrobial cold plasma which causes minimum damage to the nutritional properties and physicochemical parameters in food products^20,21^. Many studies have shown the antimicrobial potential of cold plasma on various microorganisms^19,22–25^. So far, some studies have hired cold plasma for inactivating bacteria in fruit juices^22,26,27^. Liao et al*.*, Wang et al*.*, and Xiang et al*.* investigated the antimicrobial activity of cold plasma on apple juice contaminated with *Escherichia coli* and *Zygosaccharomyces rouxii*, respectively. Liao et al*.* microbiological assay showed 4.3 log CFU/ml of reduction and Wang et al*.* and Xiang et al*.* observed 5–6.8 log CFU/ml of reduction. The effect of plasma treatment on apple juice was investigated in the mentioned studies, a loss of some antioxidants and phenolic compounds such as vitamin C and no significant change in other nutrients was reported^28–30^. Hou et al*.*^31^ studied the antimicrobial influence of non-thermal plasma on blueberry juice against *Bacillus* sp. and reported 7.2 log CFU/ml of reduction in total with low cost and loss of quality. Mahnot et al*.*^32^ successfully inactivated *Salmonella Typhimurium* in coconut water by 5 log CFU/ml of reduction using high voltage atmospheric cold plasma with minimum changes in nutritional content. A commercialized and optimistic nonthermal food processing technique should achieve effective microbial inactivation and minimal loss of quality. Still little is known about the true nature of the RONS's role in inactivating microorganisms; In fact, today’s main concern in plasma food processing is to find the most effective RONS in microorganism inactivation since it can lead to more accurate and reliable inactivation at lower treatment times.

Our aims in this study were to (1) design and optimize CAP devices for food processing in industries to increase food safety and quality; (2) investigate the influence of CAP treatments on the conventional physicochemical properties of fresh pineapple juice (FPJ); (3) evaluate the concentration of the RONS in treated FPJ samples; also (4) establishing an innovative strategy of plasma treatments for reliable and rapid disinfection of fruit juices.

## Materials and methods

### CAP treatments

The new strategy implemented by us in this study consisted of combining atmospheric pressure cold plasma generators (the plasma jet and the SDBD) with cold vapors of hydrogen peroxide (H_2_O_2_) and water (H_2_O) to increase the production level of RONS species. In this study, we investigated the effects of plasma-induced species on the bactericidal properties of the liquid medium (pineapple juice) and its physicochemical properties. Due to the above considerations, a surface dielectric barrier discharge (SDBD) structure, as well as a jet in the after-glow region, have been used^33^. The following is a description of the plasma treatment and the details of the structures used in this study.

**Figure 1:**
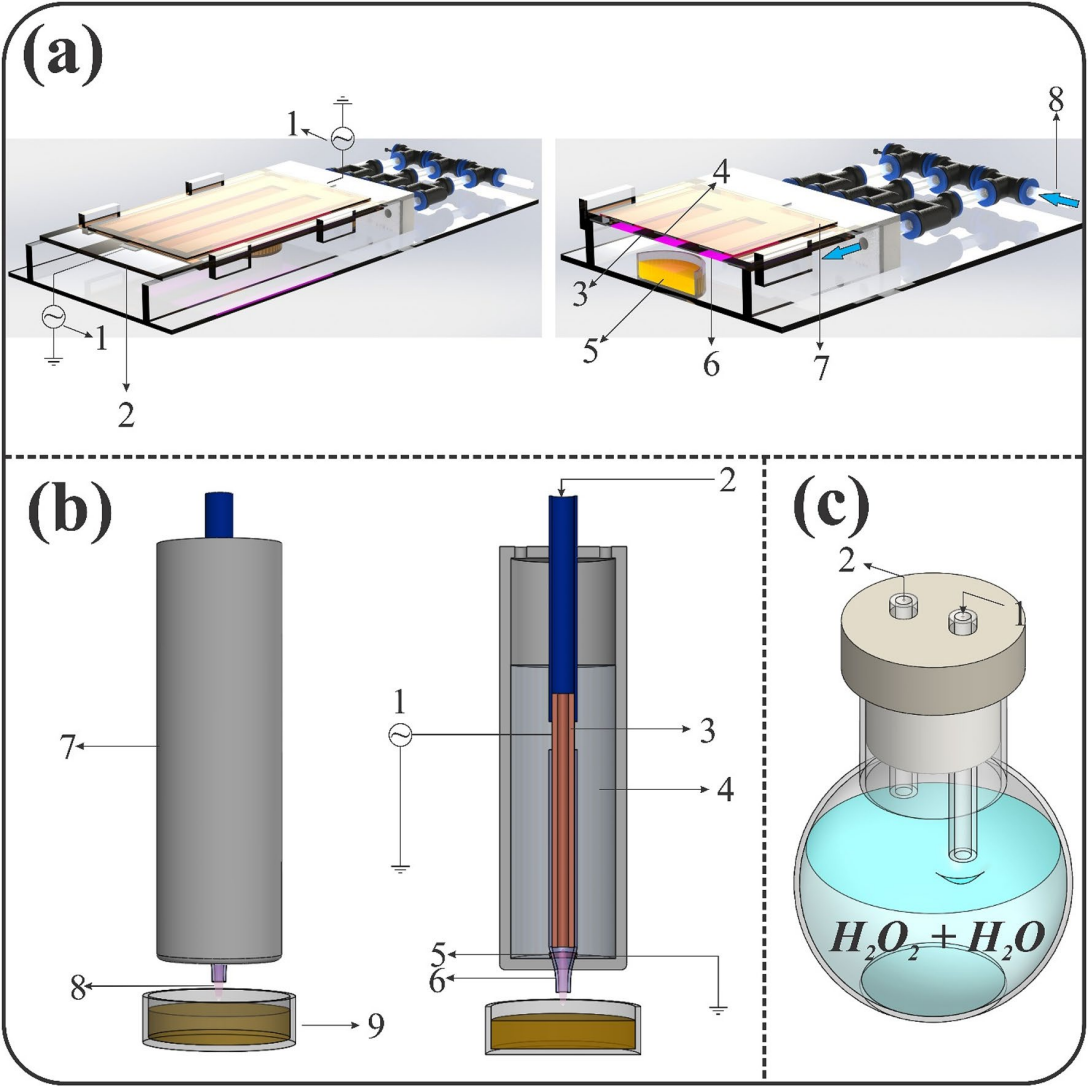
Schematic diagram of (**a**) SDBD-CP. 1: high voltage power supply, 2: dielectric barrier, 3: lower electrode, 4: upper electrode, 5: sample, 6: plasma discharge, 7: Kapton Tape, 8: gas inlet. (**b**) Plasma jet. 1: high voltage power supply, 2: gas inlet, 3: high-voltage electrode, 4: epoxy resin, 5: ring electrode, 6: quartz dielectric nozzle, 7: Teflon (PTFE) tube, 8: plasma plume, 9: sample. (**c**) Cold vapor section 1: argon gas inlet, 2: gas outlet.

The SDBD and the plasma jet structures were shown schematically in Fig. 1. The SDBD structure was depicted in Fig. 1a. In this plasma source, an electrode arrangement consisting of aluminum adhesive tapes glued edge to edge on the top and back surfaces of a 4 mm thick glass dielectric. The dimensions of the aluminum strips were 2 cm by 18 cm and spaced 2 cm apart. The applied voltage on the electrodes was 12 kV AC with 6.2 kHz. On the upper surface of the SDBD structure, wide and thin strips of Kapton tape were glued as a strong dielectric to prevent corona and loss of electrical power. The power of the SDBD was 4.2 watts. Pure argon gas (99.999%) was used as the carrier gas to transfer H_2_O_2_/H_2_O vapors from the hydrogen peroxide-water mixture bottle into the plasma reactor for both sources (see Fig. 1c). The mole fraction of H_2_O_2_ in water was $$ M_{{H_{2} O_{2} }} /M_{{H_{2} O}}   = 0.3$$ in the liquid phase and that of vapor mixture was evaluated to be $$ M_{{H_{2} O_{2} }} /M_{{H_{2} O}}   = 0.034$$ in the mixture of argon, hydrogen peroxide, and water vapors. The difference in heat of vaporization of the hydrogen peroxide and water led to a significant decrease in the mole fraction of H_2_O_2_ concerning the liquid phase. Following Giguère and Maass^34^, the total vapor pressure of the hydrogen peroxide and water was assumed to be 20 Torr at 30 °C. The partial pressure of H_2_O_2_ and H_2_O was estimated to be 0.65 Torr and 19.35 Torr, respectively. Regarding these partial pressures at 30 °C, the concentrations for H_2_O_2_ and H_2_O vapors were obtained to be 856 and 25,492 ppm (part per million), respectively. These estimations determine the upper limits for mole fractions of hydrogen peroxide and water vapors based on Giguere and Maass's findings at 30 °C. The argon gas flowing over the mixture with 4 slm (standard liters per minute) carried the vapors from the liquid bottle into the gas discharge zone (see Fig. 1a, c). A mass flow controller (Seven stars D07-19B) and a flow readout box (Seven star D08-1F) were used to adjust and read the gas flow. The plasma jet also consisted of a copper tube as the powered electrode, a quartz glass tube as the dielectric barrier, and a ring electrode as the ground wrapped around the outer surface of the quartz (see Fig. 1b). Argon gas with H_2_O_2_/H_2_O vapors flows through the inner tube into the electric gas discharge gap. The whole structure was placed inside the epoxy resin as a high-aptitude electrical insulator to avoid the occurrence of an electric spark. The outer body of the jet structure was made of PTFE (Polytetrafluoroethylene). Frequency, peak voltage, and power for the plasma jet structure were 4.7 kHz, 14.4 kV, and 4.2 W, respectively. For both plasma sources, the plasma power was considered to be the same to compare the performance of the two devices under similar conditions. In all experiments, the ambient temperature and relative humidity were constant at 23 °C and 70%, respectively. The treatment time for the structures used in this study was 30, 90, 180, and 300 s, with the difference that for the SDBD structure, the sample was also exposed to plasma for 7 min. Juice samples were placed in a petri dish and each treatment was performed in triplicate. The surface of the samples (pineapple juice) is placed at a distance of 1 cm from the conical tip of the dielectric nozzle in the plasma jet structure and also at a distance of 2 cm from the lower electrode in the SDBD structure. The gas temperature of plasma was 50 °C for the SDBD device and 32.5 °C for the jet device. All treatments with both devices were also performed without plasma to ensure the contribution of plasma to the inactivation of bacteria and every other effect.

### Preparation of pineapple juice

To prepare fresh and uninfected pineapple juice, five pineapples were collected from the same plantation. After being washed up they were skinned and squeezed in a biological safety cabinet class III (LAMSYSTEM BMB-III-Laminar-S PROTECT 1E-C.001-120). Pulps and other unwanted particles were carefully removed by performing a simple filtration under the safety cabinet. The volume of each pineapple juice sample was 10 mL in all of our experiments. And the distance between the top of the sample and the surface of the SDBD was 2 cm. The distance between the top of the sample and the tip of the jet device was 1 cm, too.

### Preparation of bacterial strain

The standard strain of *E. faecalis* (ATCC 29212) was cultured on Mueller–Hinton Agar (Ibresco i23118) and placed in an incubator at 37 °C for 24 h then transferred into the normal saline solution and made up to the concentration of 1.5 × 10^9^ CFU/ml (5 McFarland). To prepare an infected fruit juice sample for CAP treatment, 1 ml of the above-mentioned solution was added to 9 ml of juice to achieve the final concentration of 1.5 × 10^8^ CFU/ml (0.5 McFarland).

### Microbiological analysis

To present an accurate Log chart, the untreated and CAP treated samples were serially diluted in 0.9% normal saline solution. Then, 0.1 ml of each diluted solution was plated on a Mueller–Hinton agar medium culture and incubated at 37 °C for 24 h.

### Determination of organic content

Organic acids such as citric acid and malic acid, vitamin C, and sugars such as glucose, fructose, and sucrose were determined before and after the CAP treatment by reverse phase-high performance liquid chromatography (HPLC) procedure described by Tyagi, et al.^35^. All measurements were repeated three times.

#### Determination of vitamin C

The concentration of vitamin C in treated and untreated samples was determined using a Phenomenex Luna C18 column and a mobile phase of acetonitrile and 10 mM potassium dihydrogen orthophosphate buffer (40:60, pH = 2.1). The mobile phase was gently released with the flow rate of 1 ml/min and the column temperature was fixed at 50 °C. Detections were done with a UV detector set at a wavelength of 268 nm.

#### Determination of organic acids

In the case of citric acid and malic acid, the separation was performed using a Phenomenex Luna C18 column associated with the mobile phase of ammonium di-hydrogen orthophosphate (NH_4_H_2_PO_4_) buffer (2% w/v, pH = 2.18) and the flow rate of 1 ml/min. The temperature was stabilized at 32 °C. The detection of both organic acids was done by a UV–Vis detector set at a wavelength of 214 nm.

#### Determination of sugars

A binary gradient elution Phenomenex Luna NH2 column with the mobile phase of acetonitrile and water (75:25 v/v) and a flow rate of 1 ml/min was used for the separation of sugars. The column temperature was 40 °C. detections were done using a refractive index detector.

### pH, temperature, and total dissolved solids (TDS) measurements

The pH and temperature of treated and untreated samples were measured immediately after the treatment times were finished. Both pH and temperature measurements were done at the same time using an AZ 86502 pH and thermometer. Total dissolved solids (TDS) measurements were also been taken care of by a NEWCON digital TDS meter.

### Colorimetric measurements

Color is another important physicochemical factor in fruit juice. The color parameters of treated and untreated samples were measured by a hunter lab colorimeter (ColorFlex, Hunter Associates Laboratory, Inc., Reston, 232 Virginia, USA) in the CIE LAB space. Color conditions of samples were quantified as L* (whiteness/darkness), a* (redness/greenness), and b* (yellowness/blueness). Total color difference (*ΔE*) in samples after CAP treatment was shown as follows:

$$  \Delta E = \left( {\left( {L_{0}  - L_{i} } \right) + \left( {a_{0}  - a_{i} } \right) + \left( {b_{0}  - b_{i} } \right)} \right)^{{1/2}}   $$

The letters with subscript 0 are the color values of the samples before and the letters with subscript i are the color values of treated samples.

### RONS measurements

#### ROS

The concentration of H_2_O_2_ in the liquid sample was measured directly by UV/Vis spectroscopy using a reagent solution of potassium titanium(IV) oxalate dihydrate in H_2_O/H_2_SO_4_^36^. Despite the H_2_O_2_, other ROS radicals cannot be measured directly in water solution due to their short-living nature. Other radicals do not exist long enough to be measured by reagents; hence a special group of compounds known as spin traps must be hired to scavenge these ROS and form a spin trap-radical complex which can keep long enough to be measured by an electron paramagnetic resonance (EPR)^37^. The concentration of hydroxyl radical (^·^OH), ozone (O_3_)/atomic oxygen (O), and hydroperoxyl (^·^OOH) were measured by the EPR method with thanks to spin traps. PBS solutions of spin traps 2,2,6,6-tetramethylpiperidine (TEMP, ≥ 99%), 5,5-dimethyl-1-pyrroline *N*-oxide (DMPO, ≥ 98%) and 5-(diethoxyphosphoryl)-5-methyl-1-pyrroline *N*-oxide (DEPMPO, ≥ 99%) were prepared at the concentration of 0.1 M, ready to be used. 0.1 M solution of singlet oxygen (O_2_(a^1^∆g)) scavenger sodium azide (NaN_3_, ≥ 99%) was added to TEMP to make difference between the amount of 2,2,6,6-tertramethylpiperidine 1-oxyl (TEMPO) formed from O_3_/O and O_2_^38,39^. An aqueous solution of 5-(2, 2-dimethyl-1, 3-propoxycyclophosphoryl)-5-methyl-1-pyrroline N-oxide (CYPMPO) and phenyl-*tert*-butyl nitrone (PBN) were used to scavenge superoxide radical anions (O_2·_^_^)^40,41^. In a typical experiment, PBS and water solutions containing spin traps were suspended in fresh pineapple samples by shaking them roughly at − 16 °C for 21 h. After preparations, spin traps containing samples were exposed to each plasma device for 30–420 s.

EPR measurements and detections were done with a Magnettech MiniScope MS 200 spectrometer. Required parameters including frequency, modulation frequency, modulation amplitude, power, sweeping time, and sweeping width were precisely adjusted according to the research of Privat-Maldonado et al.^39^, and all scans were repeated three times. Analyzed samples were prepared in 50 µL glass capillaries for measurements. The results were procured by double integration (SpectrumViewer ver. 2.6.3) of the respective simulated spectra (NIH P.E.S.T. WinSIM software ver. 0.96) of the formed radical adducts.

#### RNS

Most RNS consists of nitrate and nitrite anions which are long-lived reactive species. Both nitrate and nitrite radicals were measured using ion chromatography columns. Cartridge Strata® C18-E was used to omit organic molecules from the samples to ensure accurate evaluation by the ion chromatography method. Column Metrosep A Supp 10—250/4.0 was hired with anion eluent composition of 5 mM Na_2_CO_3_ + 5 mM NaHCO_3_ and flow of 1 ml/min and pressure level of 14.94 MPa. Metrohm-model 881 compact IC pro 1 was hired with a recording time of 36 min and manual integration to detect the concentration of nitrate and nitrite in treated and untreated samples.

### Statistical analysis

Each experimental condition, preparation, and requirement was performed in triplicate and each experiment was separately repeated three times. Data are presented as mean ± standard deviation (SD). Differences among groups are also reported using the statistical package SPSS 24.0 (IBM, Armonk, NY, USA). The chosen procedure for comparing the data was one-way ANOVA combined with Duncan’s multiple comparison test at a 95% confidence level and a significance level of *p* < 0.05.

## Results and discussions

### Plasma-induced H_2_O_2_ and generation of RONS in the pineapple juice

**Figure 2:**
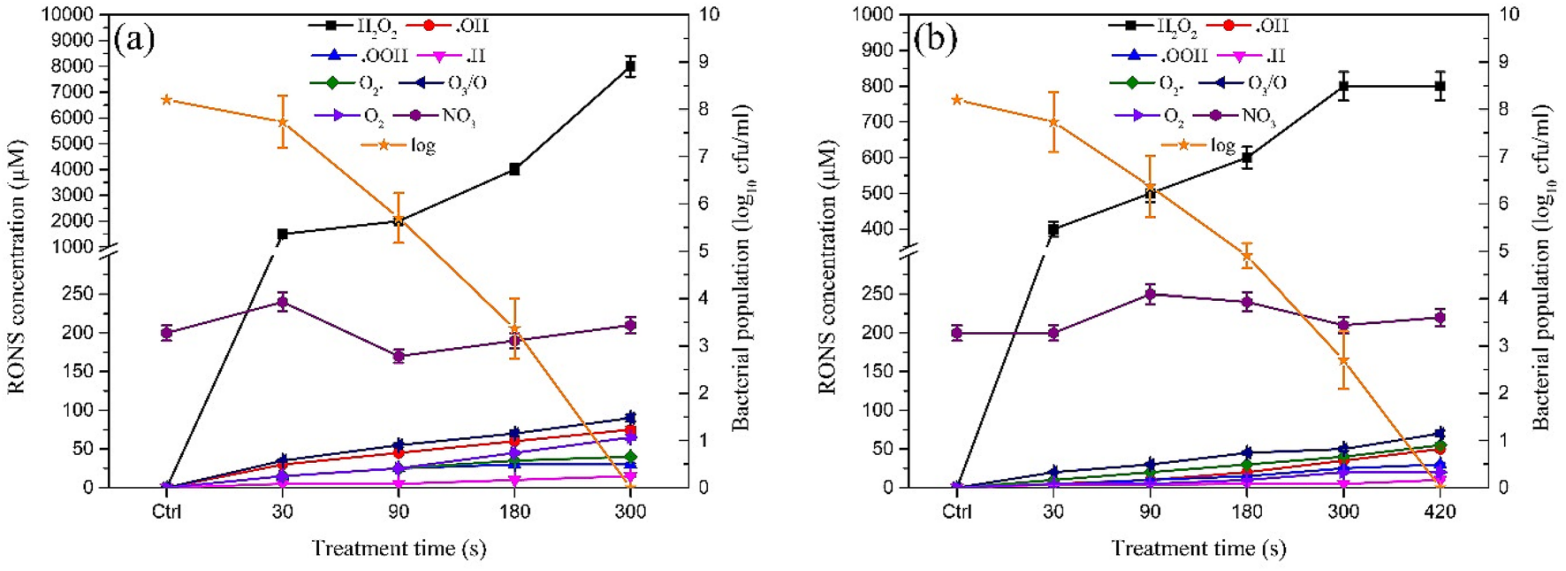
Time evolution of the concentration of RONS during treatments with plasma jet (**a**) and SDBD (**b**). The remaining log population diagram has also been involved for a comparison between log reduction and the amount of each RONS in treated samples.

Evaluating the induced concentration of the ROS in the juice for both CAP devices is essential to clarify which one of them could be more responsible for the inhibition of the bacteria described above. As shown in Fig. 2, the levels of H_2_O_2_ in SDBD treated samples were significantly lower than the plasma jet and the increasing rate of the H_2_O_2_ concentration during the treatment in plasma jet was faster than SDBD. The H_2_O_2_ concentration after SDBD treatment for 30 s was 400 μM (13.60 ppm), which had risen to 800 μM (27.21 ppm) with treatment time to 300 s and remained 800 μM with the treatment time of 420 s. In the case of the plasma jet, the concentration of H_2_O_2_ was 1500 μM (51.02 ppm) after 30 s and increased rapidly to 8000 μM (272.10 ppm) after 300 s. Besides H_2_O_2_, ROS radicals such as superoxide, hydroxyl, ozone, atomic oxygen, and hydroperoxyl were also generated in plasma and induced in the FPJ during the treatment times from 30 to 420 s. Evaluating the concentration of ROS radicals with spin-trapping and EPR showed that plasma jet induces more ROS into the samples than SDBD, nevertheless, the increase of concentration was observed during the treatment times in both devices. As shown in Fig. 2 excluding the hydrogen peroxide, the concentration and increasing rates of ozone and atomic oxygen were higher than other ROS in both plasma devices.

Despite the combination of the gas feed used, RNS are generated in plasma from the nitrogen of the environment regarding the fact that both devices were open-air^42^.

During plasma treatments with the jet, the average concentration of the nitrate was 200 μM in control samples and after 30 s it significantly increased to 250 μM but after 90 s it had a significant decrease to 170 μM; after 180 s the concentration arose significantly to 190 μM and gently increased to 200 μM after 300 s. In the case of SDBD, the average concentration of nitrate in control samples did not arise after 30 s of treatment but after 90 s it significantly increased to 250 μM but after 180 s it slightly decreased to 240 μM. After 300 s it surprisingly decreased to 210 μM and then gently increased to 220 μM.

The concentration of nitrite anions was very low in comparison to nitrate anions and out of the scope of the ion chromatography method described elsewhere.

Previous investigations have demonstrated that RONS produced by plasma during treatments with jet or SDBD may play an important role in microbial inactivation^43,44^. RONS can induce critical damages to the cell wall, cell membrane, or cellular components. Mentioned changes can inactivate the spoiling microorganisms make it impossible for them to recover^2,45^.

### Plasma device and treatment time-dependent inactivation of *E. faecalis*

Populations of *E. faecalis* in FPJ Before and after plasma jet and SDBD treatments are shown in Table 1, treatments with SDBD and jet disabled *E. faecalis* in FPJ samples in a time and device-dependent manner. No significant change was observed after 30 s of treatment with both devices but after reaching the time of 90 s the population started to reduce significantly. Finally, 8.2 log reduction *E. faecalis* was accomplished by treating samples with SDBD at 4.2 W for 420 s, while the same reduction was observed by exposure of jet at 4.2 W for 300 s. Treating samples without plasma but under the same conditions for the same times did not cause sufficient disinfection. These results strongly indicate that SDBD and jet can remarkably eliminate *E. faecalis* from FPJ but in different treatment times.

**Table 1:** Log (CFU/ml) of the bacterial population in untreated samples (Ctrl) and treated samples with plasma jet and SDBD at different treatment times.

Samples	Log (cfu/ml)
Ctrl	8.20 ± 0.00^a^
Jet 30ʺ	7.73 ± 0.55^a^
Jet 90ʺ	5.70 ± 0.53^b,c^
Jet 3ʹ	3.36 ± 0.63^d^
Jet 5ʹ	0 ± 0.00^e^
SDBD 30ʺ	7.73 ± 0.63^a^
SDBD 90ʺ	6.37 ± 0.64^b^
SDBD 3ʹ	4.90 ± 0.26^c^
SDBD 5ʹ	2.70 ± 0.61^d^
SDBD 7ʹ	0 ± 0.00^e^

^a^Significant difference (*p* < 0.05) within each column was determined by different superscript letters.

^b^Values are means ± SD (n = 3).

To show that the presence of plasma had a great contribution to the disinfection of the pineapple juice, all the microbiological tests were performed without the plasma but at the presence of the gas flow of Ar/H_2_O_2_/H_2_O. The absence of plasma was the only difference between gas flow treated samples and plasma-treated samples and all other experimental conditions were set the same as with the plasma treatment. The remaining bacterial population of each sample was counted after the end of its treatment time. The results of gas flow treated samples can be seen in Table 2. It can be concluded from Table 2 that total disinfection can’t be achieved by gas flow treatment at the mentioned times. The statistical analysis of the results showed that treating the samples for 90 s with both devices had no significant effect on the bacterial population in the samples.

**Table 2:** Survived bacteria after treatment by the gas flow of Ar/H_2_O_2_/H_2_O without running plasma.

Samples	Log (cfu/ml)
Ctrl	8.20 ± 0.00^a^
Jet 30ʺ	8.10 ± 0.05^a^
Jet 90ʺ	7.90 ± 0.10^a^
Jet 3ʹ	6 ± 0.26^d^
Jet 5ʹ	4.44 ± 0.32^f^
SDBD 30ʺ	8.13 ± 0.11^a^
SDBD 90ʺ	7.93 ± 0.11^a^
SDBD 3ʹ	7.53 ± 0.15^b^
SDBD 5ʹ	6.50 ± 0.30^c^
SDBD 7ʹ	5.06 ± 0.20^e^

^a^Significant difference (*p* < 0.05) within each column was determined by different superscript letters.

^b^Values are means ± SD (n = 3).

To show that H_2_O_2_ is not the only responsible RONS for the bactericidal activity, we checked the bactericidal capability of H_2_O_2_ by adding pure H_2_O_2_ to the samples. As shown in Fig. 2 the highest concentration of H_2_O_2_ during our treatments with plasma jet was 8000 µM and that of the SDBD treatments was 800 µM. We prepared the mentioned concentrations with pure H_2_O_2_ and fresh pineapple juice. After preparing each solution the bacterial population of 0.5 McFarland (8.2 log) was immediately added to each solution and shook three times gently. The enumeration of the remaining bacterial population was performed 60 s after the addition of 0.5 McFarland. All of the mentioned procedure was replicated three times. The population of bacteria in the H_2_O_2_ added pineapple juice has been reduced to 1.33 ± 0.15 log after the addition of 8000 µM H_2_O_2_. And the addition of 800 µM of H_2_O_2_ reduced the bacterial population to 5.06 ± 0.49 log. This experiment showed that neither any of the 8000 µM and 800 µM concentrations of H_2_O_2_ can eradicate the bacterial population of infected samples, therefore the H_2_O_2_ is not lonely responsible for the bactericidal potential of our cold atmospheric plasma strategy.

### Influences of CAP treatments on conventional physicochemical properties of FPJ

The main physicochemical characters of our FPJ samples, including pH, temperature, and total dissolved solids (TDS) were profiled and measured before and after plasma treatments. As shown in Table 3, the average pH of control samples was 3.90 by 300 s of exposure to plasma by jet, the average pH increased slowly to 3.96. The same increase of pH was observed after 420 s of treatment with SDBD. The presence of ROS and NO_x_ anions may cause changes in pH as was reported in some studies^30,32^. The impact of CAP treatment on TDS was not significantly changed after 90 s of treatment with plasma jet and SDBD. But referring to Table 3, the TDS of samples increased to 2047 ppm and 2070 ppm after 300 s and 420 s of plasma jet and SDBD treatments, respectively. Among pH and TDS, the temperature might also be an important parameter in food processing since it has been reported that raise in temperature can lead to the degradation of oligosaccharides in fruit juices^46,47^. The temperature of all treated and untreated samples was measured immediately after treatment times. As shown in Table 3, the temperature of control samples was equal to the room temperature (22.33 °C) and it was not significantly changed after 90 s of treatment with plasma jet but after 180 s it increased to 22.67 °C and 23.67 °C after 300 s. Treatment of samples with SDBD had no significant effect on the temperature of the samples until 300 s but after that, it increased to 23.33 °C. The treatment of juice samples with our chosen methods might have induced changes in the temperature of them but it is unlikely to cause any degradation in nutrients^47^.

**Table 3:** Physicochemical properties of food products including temperature, pH, and total dissolved solids (TDS), obtained from untreated (Ctrl) and plasma jet and SDBD treated samples at mentioned times.

Samples	Temperature (°C)	pH	TDS (ppm)
Ctrl	22.33 ± 0.58^c^	3.90 ± 0.01^d^	2003.33 ± 5.77^c^
Jet 30ʺ	22.33 ± 0.58^c^	3.92 ± 0.01^b,c^	2006.67 ± 5.77^c^
Jet 90ʺ	22.67 ± 0.58^b,c^	3.93 ± 0.01^b,c^	2003.33 ± 5.77^c^
Jet 3ʹ	22.67 ± 0.29^b,c^	3.94 ± 0.01^a^	2046.67 ± 11.55^b^
Jet 5ʹ	23.67 ± 0.58^a^	3.96 ± 0.01^a^	2046.67 ± 11.55^b^
SDBD 30ʺ	22.33 ± 0.58^c^	3.91 ± 0.01^c,d^	2003.33 ± 5.77^c^
SDBD 90ʺ	22.33 ± 0.58^b,c^	3.92 ± 0.01^b,c^	2003.33 ± 5.77^c^
SDBD 3ʹ	22.57 ± 0.11^b,c^	3.93 ± 0.01^b,c^	2043.33 ± 15.27^b^
SDBD 5ʹ	22.90 ± 0.17^a,b,c^	3.94 ± 0.01^a^	2053.33 ± 11.55^a,b^
SDBD 7ʹ	23.33 ± 0.58^a,b^	3.95 ± 0.01^a^	2070 ± 26.46^a^

^a^Significant difference (*p* < 0.05) within each column was determined by different superscript letters.

^b^Values are means ± SD (n = 3).

### Influences of CAP treatments on the color of FPJ

The color of fruit juices and beverages is usually the result of the presence of a group of macromolecules called “Carotenoids”^48^. Carotenoids are also believed to contribute to the antioxidant potential of fruit juices^49^. Three color indexes L*, a*, and b*, and the color difference from control samples ΔE were measured before and after treatment times, and this procedure was repeated three times^50,51^. The color parameters of the samples are listed in the CIE LAB color coordinates in Table 4. During the treatment times with SDBD, the amount of L* and a* were not significantly changed after 180 s but the amount of b* suddenly decreased from 23.72 to 14.08 after 30 s of treatment and it was not significantly changed after 180 s, but after that, it started to decline to 10.04. During the treatment times with plasma jet, the amount of L* and a* were not significantly changed until 180 s of treatment but the amount of b* reduced from 23.72 to 15.10 after 30 s and it declined to 9.01 by 300 s. Significant changes of L* and a* during the treatment times might be in contact with the fact that carotenoids can get degraded by free radicals and reactive species^52–54^. Regarding the concentration of ROS and RNS in our treated samples, free radicals could be responsible for the loss of the carotenoids in our treated samples. In conflict with L*, a*, and b*, the amount of ΔE was not significantly changed at all. In summary, plasma treatments with both SDBD and plasma jet device mostly changed the amount of b*, but the other color indicators were slightly declined and ΔE was not significantly changed at all.

**Table 4:** Colorimetric parameters, L*, a*, and b* and color difference, ΔE obtained from untreated samples (Ctrl) and treated samples with plasma jet and SDBD at different treatment times.

Samples	L*	a*	b*	ΔE
Ctrl	39.27 ± 0.08^a,b^	− 4.71 ± 0.06^b^	23.72 ± 0.10^a^	–
Jet 30ʺ	38.81 ± 0.05^a,b^	− 6.09 ± 0.53^c^	15.10 ± 1.03^b^	3.67 ± 0.69^a^
Jet 90ʺ	35.41 ± 2.10^b,c^	− 4.14 ± 0.01^a,b^	10.33 ± 1.86^d,e^	4.46 ± 0.21^a^
Jet 3ʹ	39.74 ± 5.34^a^	− 4.04 ± 0.88^a,b^	8.92 ± 0.89^e^	3.88 ± 1.15^a^
Jet 5ʹ	34.01 ± 1.31^c^	− 3.39 ± 1.04^a^	9.01 ± 2.34^e^	4.72 ± 1.46^a^
SDBD 30ʺ	37.18 ± 1.15^a,b,c^	− 4.81 ± 0.14^b^	14.08 ± 1.05^b,c^	3.85 ± 0.66^a^
SDBD 90ʺ	37.13 ± 0.73^a,b,c^	− 4.90 ± 0.11^b^	14.90 ± 1.56^b^	3.77 ± 0.57^a^
SDBD 3ʹ	36.45 ± 0.54^a,b,c^	− 4.71 ± 0.03^b^	14.89 ± 1.85^b^	3.85 ± 0.49^a^
SDBD 5ʹ	33.38 ± 2.50^c^	− 4.18 ± 0.45^a,b^	11.89 ± 0.82^c,d^	4.51 ± 0.50^a^
SDBD 7ʹ	34.38 ± 0.47^c^	− 3.33 ± 0.05^a^	10.04 ± 1.47^d,e^	4.48 ± 0.37^a^

^a^Significant difference (*p* < 0.05) within each column was determined by different superscript letters.

^b^Values are means ± SD (n = 3).

### Influences of CAP treatments on the organic content of FPJ

The organic content of FPJ, particularly sugars, organic acids, and vitamin C are known to have health benefits for consumers of every age, and therefore their presence is an important nutrition quality of fruit juices and beverages^55^. On the other hand, sugars and organic acids cause the sweetness and sourness of the taste respectively, thus they play a key role in the flavor of fruit juices which is commercially important for processed juices and beverages^35,56^. The concentration of nutrients in pineapple juice before and after plasma treatments is detailed in Table 5. All values are in milligrams per 100 ml. Plasma treatments made no significant difference to the concentration of vitamin C until 180 s of treatment with SDBD and 90 s of treatment with plasma jet. Almost the same manner was observed in the case of citric acid and malic acid, there was no significant difference between the concentration of citric acid by the time of 90 s of SDBD and 30 s of plasma jet and the untreated samples but after the mentioned treatment times it was slowly reduced. The concentration of malic acid did was not significantly changed after 180 s of SDBD treatment and 90 s of plasma jet treatment. In the case of sugars, fructose and glucose showed similar behavior during treatment times but the concentration of the sucrose was an exception! The average concentration of fructose was not significantly changed until 90 s of treatment with the SDBD and plasma jet, after the mentioned treatment time the concentration of the fructose reduced slowly. The average concentration of glucose was not significantly changed until 90 s and 180 s of exposure to SDBD and plasma jet, respectively. The average concentration of glucose reduced slowly after the mentioned treatment times. The indifference with glucose and fructose, the average concentration of sucrose reduced after 30 s of treatment with the SDBD and plasma jet. On the other hand, the reduction rate of sucrose was somehow higher than glucose and fructose. The reason for this phenomenon might be related to the molecular structure of sucrose; sucrose is a disaccharide made of a glucose and fructose molecule which are attached by a special kind of covalent bond known as the glycosidic linkage. The glycosidic linkage can be disrupted by ROS and hydrogen peroxide^57,58^. In short, ROS and hydrogen peroxide can turn sucrose molecules into glucose and fructose molecules and that may illustrate the difference in reduction rates between monosaccharides and sucrose.

**Table 5:** The organic content (mg/100 ml) of untreated (Ctrl) and plasma jet and SDBD treated samples at different times.

Samples	Malic acid	Citric acid	Vitamin C	Fructose	Glucose	Sucrose
Ctrl	20.63 ± 0.02^a^	57.10 ± 0.61^a,b^	0.09 ± 0.00^a^	307.30 ± 0.10^a^	313.70 ± 0.17^a^	678.67 ± 0.58^a^
Jet 30ʺ	20.62 ± 0.02^a^	57.47 ± 0.58^a^	0.09 ± 0.00^a,b^	307.23 ± 0.11^a^	313.70 ± 0.10^a^	677.33 ± 0.58^a,b^
Jet 90ʺ	20.62 ± 0.01^a^	55.77 ± 1.04^b,c,d^	0.09 ± 0.00^a,b^	307.07 ± 0.11^a,b^	313.57 ± 0.06^a,b^	674.33 ± 0.58^b,c^
Jet 3ʹ	20.55 ± 0.05^b^	56.13 ± 0.25^a,b,c,d^	0.08 ± 0.01^b,c^	306.80 ± 0.20^b,c^	313.47 ± 0.21^a,b,c^	673.67 ± 2.08^c^
Jet 5ʹ	20.42 ± 0.03^d^	54.83 ± 1.36^d,e^	0.07 ± 0.01^c,d^	306.37 ± 0.25^d,e^	313.23 ± 0.32^b,c^	668 ± 2.65^d^
SDBD 30ʺ	20.63 ± 0.01^a^	56.67 ± 0.06^a,b^	0.09 ± 0.00^a,b^	307.20 ± 0.10^a^	313.73 ± 0.06^a^	677.33 ± 0.58^a,b^
SDBD 90ʺ	20.62 ± 0.01^a^	56.47 ± 0.40^a,b,c^	0.09 ± 0.00^a,b^	307.03 ± 0.21^a,b,c^	313.57 ± 0.06^a,b^	674.67 ± 1.53^b,c^
SDBD 3ʹ	20.61 ± 0.01^a^	55.13 ± 0.93^c,d,e^	0.08 ± 0.01^a,b^	306.77 ± 0.15^c^	313.17 ± 0.21^c^	674 ± 1.00^b,c^
SDBD 5ʹ	20.56 ± 0.04^b^	54.70 ± 0.17^d,e^	0.08 ± 0.01^b,c^	306.47 ± 0.06^d^	313.10 ± 0.10^c^	671.67 ± 2.08^c^
SDBD 7ʹ	20.49 ± 0.03^c^	54.23 ± 1.07^e^	0.07 ± 0.01^d^	306.13 ± 0.15^e^	312.47 ± 0.42^d^	665.67 ± 4.04^d^

^a^Significant difference (*p* < 0.05) within each column was determined by different superscript letters.

^b^Values are means ± SD (n = 3).

## Conclusion

In this study, the efficiency of reinforced plasma jet and SDBD by H_2_O_2_ and H_2_O cold vapors on the inactivation of *E. faecalis* in FPJ was investigated. The results showed that exposure of plasma jet and SDBD at 4.2 W for 300 s and 420 s respectively, resulted in an 8.2 log reduction of *E. faecalis* cells in FPJ. Both plasma jet and SDBD induced a remarkable deal of ROS and RNS in treated samples resulting in a log reduction of *E. faecalis*. Some reactive species such as H_2_O_2_ had higher concentrations than others and are believed to have more contribution in inactivating bacteria cells and the following log reduction since a comparison between the log reductions and the concentration of RONS in both plasma devises shown in Fig. 2 indicates that the reduction of log and the increase of ROS especially H_2_O_2_ happened simultaneously.

Gentle decreasing rate on some of the organic content of the FPJ and minimal damage to them supports the fact that the plasma caused damage to the organic content of FPJ in both devices was negligible. Besides, plasma treatments caused adverse effects on color characteristics of FPJ samples, and no significant effect on the color difference (ΔE) between control and treated samples.

In summary, our results and findings explicitly suggest that our rapid innovative strategy of plasma treatments can effectively inactivate *E. faecalis* (8.2-log CFU/ml) in FPJ with minimal damage to its quality and no addition of harmful materials. In the future, studies and investigations should focus on proposing optimized, low-cost, and scale-up plasma procedures or devices for wide applications in food industries. It was also concluded that although the SDBD enhanced by H_2_O_2_/H_2_O has relatively less disinfectant power, due to its large scale, it can promise great potential for disinfection of fruit juices in line with commercial plans.
